# Incident HIV during pregnancy and early postpartum period: a population-based cohort study in a rural area in KwaZulu-Natal, South Africa

**DOI:** 10.1186/s12884-017-1421-6

**Published:** 2017-07-26

**Authors:** Terusha Chetty, Alain Vandormael, Claire Thorne, Anna Coutsoudis

**Affiliations:** 1 Africa Health Research Institute, KwaZulu-Natal, South Africa; 2 Department of Public Health Medicine, School of Nursing and Public Health, University of KwaZulu-Natal, Durban, KwaZulu-Natal South Africa; 3 KwaZulu-Natal Research Innovation and Sequencing Platform (KRISP), College of Health Sciences, University of KwaZulu-Natal, KwaZulu-Natal, South Africa; 4 UCL Great Ormond Street Institute of Child Health, University College London, London, UK; 5 Department of Paediatrics and Child Health, University of KwaZulu-Natal, Durban, KwaZulu-Natal South Africa

**Keywords:** Pregnancy, Postpartum, HIV incidence, Seroconversion, Adolescents

## Abstract

**Background:**

The evidence on the effect of pregnancy on acquiring HIV is conflicting, with studies reporting both higher and lower HIV acquisition risk during pregnancy when prolonged antiretroviral therapy was accessible. The aim of this study was to assess the pregnancy effect on HIV acquisition where antiretroviral therapy was widely available in a high HIV prevalence setting.

**Methods:**

This is a retrospective cohort study nested within a population-based surveillance to determine HIV incidence in HIV-uninfected women from 15 to 49 years from 2010 through 2015 in rural KwaZulu-Natal. HIV incidence per 100 person-years according to pregnancy status (not pregnant, pregnant, to eight weeks postpartum) were measured in 5260 HIV-uninfected women. Hazard ratios (HR) were estimated by Cox proportional hazards regression with pregnancy included as a time varying variable.

**Results:**

Overall, pregnancy HIV incidence was 4.5 per 100 person-years (95% CI 3.4–5.8), higher than non-pregnancy (4.0; 95% CI 3.7–4.3) and postpartum incidences (4.2 per 100 person-years; 95% CI 2.3–7.6). However, adjusting for age, and demographic factors, pregnant women had a lower risk of acquiring HIV (HR 0.4; 95% CI 0.2–0.9, *P =* 0.032) than non-pregnant women; there were no differences between postpartum and non-pregnant women (HR 1.2; 95% CI 0.4–3.2; *P* = 0.744). In models adjusting for the interaction of age and gravidity, pregnant women under 25 years with two or more pregnancies had a 2.3 times greater risk of acquiring HIV than their older counterparts (95% CI 1.3–4.3; *P* = 0.008).

**Conclusions:**

Pregnancy had a protective effect on HIV acquisition. Elevated HIV incidence in younger women appeared to be driven by those with higher gravidity. The sexual and biological factors in younger women should be explored further in order to design appropriate HIV prevention interventions.

## Background

In sub-Saharan Africa, HIV risk is greatest among women of reproductive age [1], with relatively high HIV-1 incidence (0–16.8 events per 100 person-years) during pregnancy and the immediate postpartum period [2]. Evidence is conflicting on the pregnancy effect on HIV acquisition; in Uganda, Zimbabwe and Rwanda, pregnancy increased HIV susceptibility [2–5]. However, in a pooled analysis of six African cohorts, up to 2011, pregnant women had lower HIV acquisition risk than non-pregnant women due to greater concordance with sexual partners during pregnancy versus non-pregnancy [6].

Fertility has declined gradually to 3.5 children per woman among Black South Africans versus 3.2 children per woman nationally [7]. The fertility decline was typified by childbearing postponement and longer birth intervals, due to contraceptive accessibility since the 1970s [8]. While fertility in older women is stabilizing, high adolescent fertility rates (65 per 1000 women aged 15–19 years) [7, 9] and HIV incidence (17.2 per 100 person-years) [10] are consequences of unprotected sexual intercourse. Young South African women have an 80% lifetime HIV risk [11]. HIV incidence in 15–26 year olds with early adolescent pregnancies in Eastern Cape was 6.0 per 100 person-years [12]. In the pooled analysis mentioned earlier, the pregnancy protective effect on HIV risk was not observed in 15–24 year olds [6]. While there are challenges around pre-exposure prophylaxis (PrEP) use in younger women [13], high HIV incidence in this population highlights the necessity for effective, accessible biomedical interventions.

Women have greater HIV risk with larger mucosal surface areas exposed to infectious fluids and pathogens and potentially more tissue damage during intercourse [5]. Young women are particularly vulnerable to HIV, with cervical ectopy enhancing target cell exposure to trauma and vaginal pathogens [5]. Pregnancy may also facilitate HIV infection through hormonal changes, genital mucosal alterations and sexual behaviour of women and their partners [3, 14, 15].

South Africa introduced the World Health Organization Option B [16] in 2013 and in 2015 adopted Option B+ and a CD4^+^ cutoff of 500 cell/mm^3^ for treatment of HIV-infected patients [17]. In 2016 all HIV-infected patients irrespective of CD4^+^ count became eligible for antiretroviral therapy (ART) [18]. Globally, South Africa has the largest number of people on ART, with approximately 45% of HIV-infected people [95% confidence interval (CI) 43%–49%] on ART in 2014 [19, 20]. In KwaZulu-Natal (KZN), HIV incidence fell by 37% after ART coverage increased from <10% to ≥40% [11]. While ART coverage has improved, potentially protecting uninfected partners from HIV [11], few studies have reported lower HIV risk in pregnancy.

The study objective was to explore pregnancy effects on HIV acquisition during widespread ART availability in rural South Africa. A secondary objective was to present HIV incidence trends by pregnancy status, age and year.

## Methods

Data from a retrospective cohort of women aged 15–49 years nested within a population-based surveillance were analysed.

### Setting

Since 2000, the Africa Centre has collected demographic and health data through longitudinal surveillance of approximately 90,000 resident and non-resident members in 12,000 households in the Hlabisa sub-district of uMkhanyakhude [21]. The 2011 HIV prevalence was 38% among females versus 16% in males 25–29 years [22]. HIV incidence peaked at 6.6 per 100 person-years in 24 year old women between 2004 and 2011 [11]. The 2011 ART coverage for 25–49 year old females was 40%, higher than their male counterparts at 30% [22].

### Household and individual survey eligibility

Surveillance eligibility includes household members, who may be resident or non-resident members [21]. Residency is defined as living at a physical structure within the surveillance area at a particular time-point [21]. The inclusion of non-resident members is necessary to understand HIV transmission patterns as non-residents may be subject to differing HIV risk compared to residents [23]. Two surveys are conducted by interview: (i) household surveillance (initially biannual, since 2012 three times per year); and (ii) an annual individual resident survey, including optional individual HIV surveillance for residents’ ≥15 years.

During household surveys, a key proxy respondent answers questions about the physical structure, households, individuals and their relationships. New pregnancy data (for resident and non-resident women) are routinely collected at every household survey, with a pregnancy notification form completed by the proxy respondent (who may be a pregnant woman). Pregnancy outcomes are recorded in the pregnancy notification form during proxy respondent interviews at subsequent rounds. Formal pregnancy testing is not conducted in the surveillance area.

Data on prior pregnancies in women of reproductive age (15–49 years) are only collected in individual resident surveys, and not collected by proxy in household surveys. Hence, a perpetually non-resident woman will lack data on prior pregnancies; a current pregnancy will be notified through a pregnancy notification form as above.

The survey intervals mean that most pregnancies in a household should be ascertained, although under-ascertainment of early miscarriages is likely, as pregnancies are frequently not reported until after the first antenatal visit and/or pregnancy is not disclosed to the household proxy respondent until after the first trimester. Since 2000, there has been an average of 2200 live-births annually in the surveillance [24], with overall, stable fertility rates at about three children per woman [25].

### Identifying pregnancy episodes

Pregnancy episodes began at the last menstrual period and ended at delivery. For women with miscarriages or pregnancy termination, the estimated delivery date reported to the surveillance was used to signify the pregnancy end. All pregnancy episodes to 15–49 year old women in the surveillance, including repeat pregnancies, from 01 January 2010 to 31 December 2015 were included. Some pregnancies were ongoing at the end of December 2015.

Live births were defined as infants delivered alive at ≥28 weeks’ gestation, regardless of early neonatal death, and still births as infants not alive on delivery (no movement, crying or breathing) at ≥28 weeks. Miscarriages were pregnancies ending at <28 weeks. Induced abortions were intentional pregnancy termination either by medical abortion before 20 weeks or through self-termination.

### Identification of the outcome measure

The outcome measure was time to HIV seroconversion for repeat testing women. Repeat testers had at least two HIV tests, the first of which must have been negative to allow observation of any seroconversion, and were tested for HIV in the surveillance through 31 December 2015. In order to infer HIV negative status on the 1 January 2010, only those with their HIV negative test after 2008 were included. Survival time of HIV-uninfected women began on 1 January 2010 as the pregnancy exposure status started on this date. Follow-up time started from date of the first negative test, until exit at the last test date or seroconversion, if earlier. As the interval between HIV tests was more than a full gestation pregnancy, actual seroconversion time was uncertain. A seroconversion date was therefore randomly imputed between the latest HIV negative test and earliest HIV positive test dates [26].

### Statistical analyses

Woman data were split into non-pregnancy, pregnancy, and eight weeks postpartum episodes. The postpartum period was based on assumptions that pregnancy biological, behavioural and hormonal effects would revert to pre-pregnancy states after approximately six to eight weeks [27, 28].

Crude HIV incidence rates and 95% confidence intervals by pregnancy status were calculated according to calendar year and age.

The relationship between pregnancy and incident HIV was assessed using Cox proportional hazards regression analysis of time to HIV. Variables considered a priori to be important, age (15–24 years; 25–49 years) and gravidity (no pregnancies, one pregnancy, two or more pregnancies), were retained in multivariable models [6, 12, 29]. Participant characteristics at last survey date, including education level (less than one year, at least primary school, secondary and beyond, and unknown), residence area (rural, peri-urban, urban), residency (resident, non-resident, non-resident prior to residency), distance from level one road, distance from nearest clinic and distance from nearest primary school, women’s HIV status knowledge (yes, no, refused), knowledge (i.e. have heard) about ART (yes, no, refused) were included as potential confounding factors. Pregnancy order was the surveillance recorded count of earlier live births, plus one for the current pregnancy (first born, second/third born, fourth born or later, none, including women never pregnant). Pregnancy order, infant number at delivery (all stillborn; singleton live born or twins, with one live born; multiple births e.g. twins, both live born, or triplets, with two live born), pregnancy outcome (abortion/stillbirth/miscarriage, singleton live birth, multiple live births, not pregnant) were time varying covariates. In univariable analysis the marginal effect of each variable on HIV acquisition hazard was assessed. Univariate variables with *P* < 0.2 were included in the multivariable models; variables with *P* < 0.05 were retained in the final model. To examine whether age modified the association between pregnancy and HIV acquisition, an interaction term for age (15–24 years; 25–49 years) and pregnancy was included.

Additional analyses were also performed on the final multivariable model: (i) redefining age into five year intervals; and (iii) testing two variables, age and gravidity, for effect modification on pregnancy and HIV acquisition. All statistical analyses were conducted using Stata 13.1.

### Ethics approval and consent to participate

Ethics approval for household (E009/00) and individual surveillance (BF233/09) is annually recertified at the University of KwaZulu-Natal Biomedical Research Ethics Committee. Either the household head or a proxy in the absence of the household head provides verbal consent for household surveillance. Consent is verbal as general demographic questions are not invasive. Written informed consent is obtained from participants in the individual surveillance, including HIV testing. Data are anonymized for privacy and confidentiality.

## Results

**Fig. 1:**
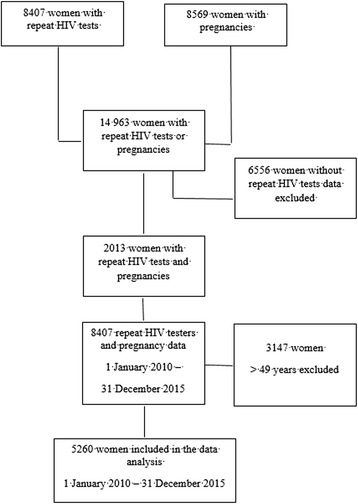
Flow diagram of study inclusions and exclusions

There were 5260 women in the study population (Fig. 1); 1621 reported pregnancies during the study. At the earliest HIV negative test, median age was 24 years (interquartile range (IQR): 19–36 years).

### Characteristics of the pregnancy exposure groups during follow-up

Women contributed 23,050 episodes: 18,095 non-pregnant episodes (78.5%), 3003 pregnancy (13.03%), and 1952 postpartum episodes (8.47%). Resident women contributed more pregnancy episodes (89.84%) than non-resident women (10.12%) (Table 1). Pregnancy was commoner among younger women (15–24 years), those with secondary and higher education, and women in rural settings. The number of pregnancy episodes with first versus second pregnancies was approximately similar and most pregnant women had reported two or more pregnancies (including the current pregnancy). Women were more likely to know their HIV status during pregnancy and postpartum episodes than in their non-pregnant state. ART knowledge was similar across groups (Table 1).

**Table 1:** Time-varying and time-invariant participant characteristics by pregnancy category

Characteristic	Not pregnant (*n* = 18,095 episodes) *n* (%)	Pregnant (*n* = 3003 episodes) *n* (%)	Postpartum (*n* = 1952 episodes) *n* (%)
Time-varying			
Age 15–24 years	7595 (41.97)	1794 (59.74)	1089 (55.79)
Time-invariant: Sociodemographic			
Episode Type^a^			
Resident	16,745 (92.54)	2698 (89.84)	1776 (90.98)
Non-resident	1281 (7.08)	304 (10.12)	175 (8.97)
Non-resident prior to residency	69 (0.38)	1 (0.03)	1 (0.05)
Home setting^a^			
Peri-urban	4971 (27.47)	849 (28.27)	547 (28.02)
Rural	11,502 (63.56)	1956 (65.13)	1282 (65.68)
Urban	614 (3.39)	81 (2.70)	59 (3.02)
Unknown	1008 (5.57)	117 (3.90)	64 (3.28)
Mean distance from level 1 road (standard deviation), km^a^	12.7 (22.0)	11.2 (18.9)	10.7 (17.6)
Maternal education^a^			
Less than one year	805 (4.45)	27 (0.90)	19 (0.97)
At least primary school	2223 (12.29)	173 (5.76)	126 (6.45)
Secondary and beyond	13,391 (74.0)	2602 (86.65)	1678 (85.96)
Unknown	1676 (9.26)	201 (6.69)	129 (6.61)
Time-varying: Pregnancy			
Pregnancy order			
First born	0 (0)	796 (40.93)	708 (40.50)
Second/third born	0 (0)	790 (40.62)	710 (40.62)
Fourth or later	0 (0)	359 (18.46)	330 (18.88)
Not pregnant	6479 (100)	0 (0.0)	0 (0)
Time-varying: Pregnancy			
Number of live born infants at delivery			
No births	18,095 (100)	31 (1.03)	19 (0.97)
Singleton	0 (0.00)	2902 (96.64)	1885 (96.57)
Multiple births	0 (0.00)	70 (2.33)	48 (2.46)
Time-invariant: Pregnancy			
Gravidity^b^			
None	2691 (14.87)	0 (0)	0 (0)
One pregnancy	4678 (25.85)	943 (31.40)	597 (30.58)
Two or more pregnancies	10,726 (59.28)	2060 (68.60)	1355 (69.42)
Time-invariant: HIV status & knowledge			
Knows HIV status			
Yes	13,780 (76.15)	2533 (84.35)	1649 (84.48)
No	3478 (19.22)	355 (11.82)	229 (11.73)
Missing	837 (4.63)	115 (3.83)	74 (3.79)
Heard about ART			
Yes	15,581 (86.11)	2669 (88.88)	1742 (89.24)
No	443 (2.45)	50 (1.67)	33 (1.69)
Missing	2071 (11.45)	284 (9.46)	177 (9.07)

^a^Characteristic quantified at the last visit

^b^Gravidity includes all pregnancies (prior and current) reported by a participant

### Incident HIV-1 infections by year and pregnancy exposure groups

There were 632 HIV seroconversions recorded over 15,711 person-years of follow-up, with an unadjusted incidence rate of 4.02 per 100 person-years. Of the 632 incident infections, 55 occurred during pregnancy (4.5 per 100 person-years; 95% CI 3.4–5.8), 11 were postpartum (4.2 per 100 person-years; 95% CI 2.3–7.6), and 566 during non-pregnancy (4.0 per 100 person-years; 95% CI 3.7–4.3).

In non-pregnant adolescents and young women, HIV incidence steadily increased from 5.03 events per 100 person-years in 2010 peaking at 11.88 events per 100 person-years in 2015 (Table 2). HIV incidence in pregnant younger women showed a more variable pattern, increasing from 4.3 per 100 person-years in 2010 to 12.2 in 2013, dropping to 5.79 per 100 person-years in 2014, then increasing again to 24.63 per 100 person-years in 2015. Notably, there were relatively wide confidence intervals after 2012. Postpartum, younger women experienced relatively fewer seroconversions compared to non-pregnant or pregnancy states (Table 3).

**Table 2:** HIV incidence during pregnancy and non-pregnancy episodes by year and age per 100 person-years (PY)

	Not pregnant				Pregnant			
	15–24		25–49		15–24		25–49	
Year	Number of seroconversion (PY)	HIV incidence (95% CI)	Number of seroconversion (PY)	HIV incidence (95% CI)	Number of seroconversion (PY)	HIV incidence (95% CI)	Number of seroconversion(PY)	HIV incidence (95% CI)
2010	105 (2089.16)	5.03 (4.15–6.09)	44 (2147.42)	2.05 (1.52–2.75)	11 (255.69)	4.30 (2.38–7.77)	0 (121.62)	0
2011	70 (1374.23)	5.09 (4.03–6.44)	51 (1813.90)	2.81 (2.14–3.70)	15 (178.99)	8.38 (5.05–13.90)	1 (109.56)	0.91 (0.13–6.48)
2012	57 (971.10)	5.87 (4.53 -7.61)	50 (1624.24)	3.08 (2.33–4.06)	6 (141.48)	4.24 (1.91 - 9.44)	1 (101.96)	0.98 (0.14–6.96)
2013	43 (641.05)	6.71 (4.97–9.04)	50 (1381.22)	3.62 (2.74–4.78)	12 (98.4)	12.20 (6.93–21.47)	2 (77.31)	2.59 (0.65–10.34)
2014	37 (400.34)	9.24 (6.70–12.76)	36 (1131.51)	3.18 (2.29–4.41)	3 (51.78)	5.79 (1.87–17.96)	2 (70.20)	2.85 (0.71–11.39)
2015^a^	16 (134.70)	11.88 (7.28–19.39)	7 (510.57)	1.37 (0.65–2.88)	2 (8.12)	24.63 (6.16–98.48)	0 (14.62)	0

^a^ Some pregnancies in the later part of 2015 would be reported in the first surveillance round in 2016 when women disclose their pregnancy to the household or attend antenatal care; hence 2015 may not include all women who were pregnant in 2015

**Table 3:** HIV incidence during the postpartum period by year and age per 100 person-years (PY)

	15–24 years		25–49 years	
Year	Seroconversion (PY)	HIV incidence (95% CI)	Seroconversion (PY)	HIV incidence (95% CI)
2010	2 (45.96)	4.35 (1.09–17.40)	2 (27.61)	7.24 (1.81–28.96)
2011	1 (36.72)	2.72 (0.38–19.33)	0 (23.92)	0
2012	2 (28.29)	7.07 (1.77–28.27)	0 (22.14)	0
2013	1 (19.78)	5.06 (0.71–35.89)	0 (19.45)	0
2014	0 (11.46)	0	1 (15.58)	6.42 (0.90–45.58)
2015^a^	1 (4.08)	24.51 (3.45–174.02)	1 (6.7)	14.92 (2.10–105.92)

^a^Some pregnancies in the later part of 2015 would be reported in the first surveillance round in 2016 when women disclose their pregnancy to the household or attend antenatal care; hence 2015 does not include all women who may have been pregnant in 2015

The HIV incidence estimates in women 25 years and older were lower in all pregnancy categories across years versus respective younger women counterparts (Table 2, Table 3).

### Models of HIV acquisition risk

The crude analysis showed no evidence of different HIV acquisition risks by pregnancy status (Table 4). There were no differences in HIV incidence by pregnancy outcome. In univariable analysis, increasing age (continuous) was protective for HIV acquisition [hazard ratio (HR) 0.94; 95% CI 0.93–0.95; *P* < 0.001] (data not shown); when age was categorized, younger women had a 2.4-fold greater hazards of acquiring HIV versus older women (Table 4).

**Table 4:** Risk factors for HIV seroconversion in women in the reproductive age group, South Africa, 2010–2015

	^a^Unadjusted hazard ratio estimates			^b^Adjusted hazard ratio estimates		
Effect	Point estimate	95% CI	*P* value	Point estimate	95% CI	*P* value
Pregnancy						
Not pregnant	Ref	-	-	Ref	-	-
Pregnant	1.1	0.8–1.5	0.420	0.4	0.2–0.9	0.032
Postpartum	1.1	0.6–1.9	0.871	1.2	0.4–3.2	0.744
Age						
25–49 years	Ref	-	-	Ref	-	-
15–24 years	2.4	2.0–2.8	< 0.001	2.5	2.1–3.1	< 0.001
Gravidity						
No pregnancies	Ref	-	-	Ref		
One pregnancy	1.4	1.1–1.9	0.006	1.5	1.1–1.9	0.004
Two or more pregnancies	1.0	0.8–1.2	0.789	1.7	1.3–2.2	< 0.001
Number of live born infants at delivery	1.02	1.0–1.03	0.004	1.02	1.0–1.03	0.033
Area of residence						
Peri-urban	Ref	-	-	Ref	-	-
Urban	0.8	0.7–1.0	0.046	0.98	0.8–1.2	0.870
Rural	0.94	0.6–1.5	0.819	1.0	0.6–1.6	0.994
Education level						
Less than one year	Ref	-	-	Ref	-	-
At least primary education	1.9	1.0–3.7	0.065	1.7	0.9–3.4	0.122
At least secondary education	3.2	1.7–5.9	< 0.001	2.0	1.1–3.8	0.035
Unknown	4.2	2.2–8.1	< 0.001	3.2	1.7–6.3	0.001
Distance to nearest level 1 road, km	1.0	1.003–1.009	0.063	0.98	0.97–1.0	0.089
Knows HIV status						
Yes	Ref	-	-	Ref	-	-
No	0.9	0.7–1.1	0.364	0.94	0.8–1.2	0.601
Refused	1.3	0.9–1.8	0.125	1.5	1.0–2.0	0.025
Age and pregnancy						
Not pregnant, 25–49 years	Ref	-	-	Ref	-	-
Pregnant; 15–24 years	2.6	1.1–6.1	0.032	2.3	1.0–5.5	0.056
Postpartum, 15–24 years	0.6	0.2–2.2	0.483	0.58	0.2–2.0	0.389

^a^Residency type, distance from level 1 clinic and nearest school, have heard about ART, pregnancy order, and pregnancy outcome were not included in the final multivariable model as *P* > 0.2 in the univariable analysis

^b^Adjusted for all listed covariates, gravidity including the most recent pregnancy

In the multivariable model, adjusting for age, and demographic factors, a statistically significant protective effect existed for pregnancy relative to non-pregnancy episodes (Table 4). Postpartum, women had a 1.2 times greater risk of acquiring HIV relative to non-pregnant women; this did not reach statistical significance. When adjusting for pregnancy and other covariates, younger women had a 2.5-fold greater risk of acquiring HIV versus older women (*P* < 0.001).

There was an interaction, by age and pregnancy, when adjusting for covariates, just reaching statistical significance (Table 4). Pregnant adolescents and young women were 2.3 times more likely to acquire HIV versus older non-pregnant women. The hazard ratios showed a slight protective effect for adolescent and younger women in the postpartum period versus older non pregnant women; this did not reach statistical significance.

The number of prior pregnancies also significantly increased HIV acquisition risk relative to no prior pregnancies. Moreover, there seemed to be a dose-response relationship between gravidity and HIV, with the hazards of acquiring HIV increasing with additional pregnancies.

Other covariates significantly associated with increased HIV acquisition included having a secondary level education relative to less than one year in school, and not knowing HIV status relative to knowledge of HIV status.

In a separate model, including an interaction term for age and gravidity and variables from Table 4, young women and adolescents with two or more pregnancies had a 2.3 times greater risk of acquiring HIV than older women with no pregnancies (95% CI 1.3–4.3; *P* = 0.008), but no increased risk was seen among those having one pregnancy. Although pregnancy relative to non-pregnancy in this model was protective, the confidence interval included 1.0 (HR 0.4; 95% CI 0.2–1.0; *P =* 0.051).

## Discussion

This study explores the population level effects of pregnancy on HIV acquisition during widespread prolonged ART availability in a high HIV prevalence rural setting. Pregnancy significantly protected against HIV acquisition, while HIV incidence postpartum was somewhat higher (not statistically significant) relative to non-pregnancy. Adolescents and young pregnant women, particularly those with two or more pregnancies, were more likely to acquire HIV than older women.

Approximately 75% of young HIV-infected people in sub-Saharan Africa are adolescents and young women between 15 and 24 years; women acquire HIV five to seven years earlier than men [30]. The 2.5 fold increased HIV acquisition risk among adolescents and young women relative to older women in our study is consistent with another KZN study [31], and in studies of women in the reproductive age group [32, 33]. In Hlabisa, the HIV incidence was highest among 24 year old females [11]. While PrEP has proven effective with good adherence [34], results from the VOICE study highlighted poor adherence among young South African women for oral or vaginal biomedical products [35]. Longer acting interventions and adherence support are necessary to comprehensive HIV strategies that adolescents and young women are able to access and control [36, 37].

Our overall HIV pregnancy incidence was similar to the pooled incidence in the meta-analysis of 19 cohorts (4.7 per 100 person-years; 95% CI 3.3–6.1 per 100 person-years) [2]. Moreover, there was a rising trend in HIV seroconversions in non-pregnant and pregnant adolescents and young women over the six years in our study.

Our findings are consistent with the ALPHA network pooled analysis from 2001 to 2011, including Africa Centre data, where pregnancy was protective against HIV infection [6]; the authors suggested this was likely due to reduced sexual activity in pregnancy and having a sero-concordant partner throughout the pregnancy. In a restricted analysis using individual Africa Centre data only, there was no difference in HIV acquisition between pregnant and non-pregnant women. The unadjusted incidence rate in our study was lower than that reported in this earlier study, overall, by pregnancy status, and the Africa Centre site [6]. This implies that women in our study may have benefitted from the transmission-reducing effects of improved ART coverage [11].

In another meta-analysis, the increased HIV acquisition risk among pregnant (pooled HR 1.3, 95% CI 0.5–2.1) and postpartum women (pooled HR 1.2, 95% CI 0.7–1.8) relative to non-pregnant/non-lactating women was not significant [2]. Postpartum findings were consistent with our results. Two of the included studies found a significantly increased HIV acquisition risk during pregnancy versus non-pregnancy [38, 39]. There may be several reasons why our study differs from prior studies [2, 3, 38, 40–42]. First, heterogeneous study populations with varying pregnancy rates and HIV prevalence, and study differences in pregnancy diagnoses, and pregnancy and postpartum classifications may complicate data interpretation. For instance, some studies reported frequent pregnancy tests [38, 41]; our study included proxy report for pregnancies which may have been misclassified, underestimating the pregnancy and postnatal HIV seroconversions. Second, some studies assessed sero-discordant couples, adjusting for reproductive health and sexual behaviours [3, 38, 41]. Our study did not assess these measures due to incomplete information, potentially biasing results away from the null.

While the South African fertility rate has stabilized or declined [7, 25], an estimated 19% of women in a population-based survey reported an adolescent pregnancy [43]. A substantial proportion of these pregnancies may be unintended within unstable relationships [44, 45]. Marston et al. found the protective effect of pregnancy on HIV acquisition did not extend to adolescents and young women under 25 years [6], while in Eastern Cape, early adolescent pregnancies were associated with a three-fold HIV risk versus non-pregnancy, as well as higher lifetime partner numbers and having a partner four or more years older [12]. We also showed that adolescents and young women with additional pregnancies had more than two-fold increased incident HIV risk than older non-pregnant women.

Overall, our findings among adolescents and young women are concerning and may reflect structural drivers influencing biomedical intervention use [10]. Emerging evidence suggests that the South African health system is failing to address adolescents’ and young women’s reproductive needs [46–48]. In response, the South African government has launched a five-pronged strategy to reduce teenage pregnancies and incident HIV in young women [49]. Young women with higher gravidity may face additional barriers due to competing childcare demands, or because they represent a marginalized group. Further research is required to explore pathways through which higher gravidity increases HIV acquisition risk. Fear of pregnancy disclosure and public stigma around teenage pregnancy may prevent early antenatal attendance and HIV service access [50].

Women with higher education attainment were likelier to acquire HIV than those with no education in our study. Though one additional education year reduced the likelihood of acquiring HIV by 7% in a prior Africa Centre study [51], a systematic review of educational attainment and HIV reported similar results, with higher education levels increasing the HIV acquisition risk, particularly in rural African areas [52].

There may be several pregnancy factors associated with HIV, including behaviour, genital mucosa alterations, and hormonal changes. Data on sexual behaviour and HIV in pregnant women or their partners are scarce. In Uganda, 1% of pregnant women reported having two or more partners in the previous year, lower than 27% of non-pregnant women, whereas 36–39% of male partners of pregnant women reported other sexual partners [3]. Progesterone elevation in late pregnancy are associated with surges in CCR5 co-receptor expression on target cells for HIV-1, potentially increasing HIV acquisition [14]. Pregnancy may also alter cervical immunity through increased pro-inflammatory cytokines levels or decreasing secretory leucocyte protease inhibitors, increasing HIV risk [15]. In young KZN women with high HIV prevalence, raised HIV target-cell recruiting chemokines and genital inflammatory cytokines were associated with HIV acquisition [53]. While there was insufficient data to assess these factors, the dose-response relationship of two or more pregnancies may signify behavioural and biological factors may contribute to the HIV vulnerability, especially in younger women [54].

Notable strengths of this study is the inclusion of one of the world’s largest HIV incidence cohorts and the ability to capture changing pregnancy status over time, allowing us to assess the effect of additional pregnancies on HIV acquisition.

### Limitations

As some pregnancies starting in late 2015 will be assessed in the first 2016 surveillance round, the 2015 pregnant seroconversions may have been underestimated. We also cannot rule out residual confounding due to inaccurate or incomplete covariate measurement, and were unable to adjust for sexual behaviour, contraception, and sexually transmitted infections (STIs). There is a demonstrated association between STIs and acquiring HIV [55–57]. In a cohort study, including Hlabisa, the baseline STI prevalence in non-pregnant HIV-uninfected women was 13% and incidence was 20/100 person-years [58]. In another KZN study, pregnancy STI prevalence was 32%; there was no difference in any STI prevalence by HIV status [59]. *C. trachomatis* and *N. gonorrhoeae* are also risk factors for pelvic inflammatory disease which may cause infertility [60]. STIs may hence modify the effect of pregnancy on HIV acquisition.

As women display well known reluctance in sexual partnership reporting, we were also unable to reliably link partner data [61]. As ART access expanded, individuals’ awareness of their HIV-infection status may have diminished participant consent in the HIV surveillance [62, 63], biasing results. This may be relevant for women tested in antenatal care. As confidence intervals after 2012 were wide in our study, more research is needed to confirm the trend of greater HIV risk in younger women and the associated factors. This study is generalizable to other rural pregnant populations in sub-Saharan Africa.

## Conclusions

This study supports prior data of the lower population HIV risk during pregnancy and early postpartum period. However, increased HIV acquisition risk in pregnant adolescents and young women in rural KZN, despite widespread ART is concerning, and adds to the debate regarding the need for PrEP in vulnerable populations. The elevated HIV incidence in younger women was associated with higher gravidity and further studies are required to understand the prenatal sexual and biological pathways that may increase HIV risk in this group to design appropriate HIV prevention interventions.

## Abbreviations

ART: Antiretroviral therapy
CI: Confidence interval
HIV: Human Immunodeficiency Virus
HR: Hazard ratio
IQR: Interquartile range
KZN: KwaZulu-Natal
PrEP: Pre-exposure prophylaxis
